# OutPredict: multiple datasets can improve prediction of expression and inference of causality

**DOI:** 10.1038/s41598-020-63347-3

**Published:** 2020-04-22

**Authors:** Jacopo Cirrone, Matthew D. Brooks, Richard Bonneau, Gloria M. Coruzzi, Dennis E. Shasha

**Affiliations:** 1 Courant Institute of Mathematical Sciences, Department of Computer Science, New York University, New York, NY 10012 USA; 2 Center for Genomics and Systems Biology, Department of Biology, New York University, New York, NY 10003 USA; 3 Center for Computational Biology, Flatiron Institute, Simons Foundation, New York, NY 10010 USA

**Keywords:** Machine learning, Computer science

## Abstract

The ability to accurately predict the causal relationships from transcription factors to genes would greatly enhance our understanding of transcriptional dynamics. This could lead to applications in which one or more transcription factors could be manipulated to effect a change in genes leading to the enhancement of some desired trait. Here we present a method called OutPredict that constructs a model for each gene based on time series (and other) data and that predicts gene's expression in a previously unseen subsequent time point. The model also infers causal relationships based on the most important transcription factors for each gene model, some of which have been validated from previous physical experiments. The method benefits from known network edges and steady-state data to enhance predictive accuracy. Our results across B. subtilis, Arabidopsis, E.coli, Drosophila and the DREAM4 simulated in silico dataset show improved predictive accuracy ranging from 40% to 60% over other state-of-the-art methods. We find that gene expression models can benefit from the addition of steady-state data to predict expression values of time series. Finally, we validate, based on limited available data, that the influential edges we infer correspond to known relationships significantly more than expected by chance or by state-of-the-art methods.

## Introduction

State-of-the-art methods for gene regulatory network inference^1–4^ use machine learning on genome-wide sequencing data to predict the interactions between transcriptional regulators and target genes. A typical approach to gene network inference is to take the results of an assay, most often binding assays such as CHIP-seq, and divide the data into training and test sets. This involves excluding some of the transcription factor-target binding observations, and using the remaining training set to infer the hidden data by some method. An issue with this approach is that it presumes that the majority of binding events are physiologically meaningful, in the sense that they influence the expression of the target gene. However, it has been shown that the physiological importance of binding can be minor^5^.

Another frequent issue with the paradigmatic network inference approach is that the resulting networks encode linear interactions (sum of weighted effects of causal elements). This modeling strategy makes pragmatic sense in the common situation in which the number of possible interactions is much greater than the experimental data points, because linear models have fewer parameters to fit^6^. Unfortunately, genomic interactions are decidedly non-linear, noisy and incomplete^7^.

For these reasons, we have approached the causality problem differently: we first attempt to build a model for each gene g that can predict the expression of that gene in left-out time points. If our model is good, then the transcription factors that most influence gene g likely constitute the causal elements for g.

The form of the model is important here. Small data sizes relative to the number of causal elements preclude the use of neural networks and, in particular, deep neural networks, which would increase the number of model's parameters. The presence of non-linear relationships excludes linear methods. As a compromise, therefore, this work uses Random Forests (RF) because they model non-linear synergistic interactions of features and perform well even when sample sizes are small^8^ though noise is always an issue.

The Random Forests within our new method OutPredict (*OP*) consist of an ensemble of regression trees tuned through extensive bootstrap sampling. We show the following: (i) The OutPredict model allows for non-linear dependencies of target genes on causal transcription factors; (ii) OutPredict can incorporate time series, steady-state, and prior (e.g. known Transcription Factor-target interactions) information to bias the forecasts; (i) OutPredict forecasts the expression value of genes at an unseen time-point better than state-of-the-art methods, partly because of steady-state and known interaction data; and (iv) the important edges inferred from OutPredict correspond to validated edges significantly more often than other state-of-the-art methods.

We compare the OutPredict method to the state-of-the-art forecasting algorithms, such as Dynamic Genie3^9^, that support forecasting and non-linear relationships, but currently lack the ability to incorporate priors. Other time-based machine learning methods such as Inferelator^6^ and Dynamic Factor Graph^10^, which we used in our previous studies^11,12^ are based on regularized linear regression. We also compare OutPredict with a neural net-based method built to predict gene expression time series^13^.

Another relevant time series method from the literature is Granger causality, which has been used successfully for small numbers of genes^14,15^. Granger causality is a vector autoregressive method that can be used to infer important transcription factors. In our case, however, we are trying to optimize predictive power using a large number of candidate transcription factors using very short time series (e.g. 6 time points). As is well known^16^, Granger causality can give misleading results in such a setting because the time series are short, causal relationships are non-linear, and the time series are non-stationary.

## Data

Public datasets vary greatly by organism with respect to experimental design, data density, time series structure and assay technologies. To show its general applicability, we test OutPredict on five different species (Table 1): (i) a Bacillus subtilis dataset (ii) an Arabidopsis dataset in shoot tissue (iii) a Escherichia coli dataset (iv) a Drosophila time series dataset, and (v) the DREAM4 one-hundred node in silico challenge. When applicable, we denote data as “gold standard“ when it is highly curated regulatory or binding data.

**Table 1:** Description of Datasets: the table shows the number of data points in each time series (in parentheses the number of replicates for each data point), available steady-state data, and the number of genes and transcription factors (TFs) under consideration for each species.

Dataset	Number of Time-points(Num of Reps)	Steady-State points	Genes	TFs	gold standard edges (TFs)
B. subtilis	7(3), 17(1), 4(3), 10(1), 10(1), 11(1), 8(1), 10(1), 11(1)^17^	52(3reps)^17^	4218	239	3144(154)^19^
Arabidopsis^12^	9(3), 9(3)	0	2173	162	1731(7)
E. coli	7(3), 7(3), 7(3), 9(3), 5(3)^20^	0	2006	163	4899(163)^9^
Drosophila	28(1)^22^	0	1000	14	1660(9)^23^
DREAM4^24^	20 different time series with 11 time-points (1rep)	201(1rep)	100	100	176(41)

“Gold standard“ data is either well-curated binding data or regulated data or both.

### B. subtilis

This dataset consists of time series and steady-state data capturing the response of B. subtilis to a variety of stimuli^17^. The gold standard network prior is a curated collection of high confidence edges from high throughput ChIP-seq and transcriptomics assays on SubtiWiki^18^ (we used the parsed data set provided in^19^).

### Arabidopsis thaliana in shoots

This dataset consists of gene expression level measured from shoots over the 2-hours period during which the plants are treated with nitrogen^12^. As gold standard network data, we used experimentally validated edges from the plant cell-based *TARGET* assay, which was used to identify direct regulated genome-wide targets of N uptake/assimilation regulators^12^.

### E. coli

This dataset includes the E. coli gene expression values, measured at multiple time points following five distinctive perturbations (i.e., cold, heat, oxidative stress, glucose-lactose shift and stationary phase)^20^. We used as gold standard ancillary data the regulatory interactions aggregated from a variety of experimental and computational methods that has been collected and described in RegulonDB^21^. We retrieved both parsed expression dataset and gold standard data from^9^.

### Drosophila melanogaster

This dataset consists of gene expression levels covering a 24-hour period; it captures the changes during which the embryogenesis of the fruitfly Drosophila occurs^22^. As gold standard network data, we used the experimentally validated TF-target binding interactions in the DroID database^23^. These interactions come from a combination of ChiP-chip/ChIP-seq, DNAse footprinting, *in vivo*/vitro reporter assays and EMSA assays across various tissues from 235 publications. Huynh *et al*.^9^ also used this Drosophila data.

### DREAM4 synthetic data

This synthetic dataset from the DREAM4 competition consists of 100 genes and 100 TFs (any gene can be a regulator)^24^. Because this is synthetic data, the underlying causality network is known.

## Methods

### Time series predictions using Random Forests

OutPredict learns a function that maps expression values of all active transcription factors at time t, to the expression value of each target gene (whether a transcription factor or not) at the next time point. Thus, for each gene target, OutPredict learns a many-to-one non-linear model relating transcription factors to that target gene.

The gene function is embodied in a Random Forest, as used previously in Genie3^25^, iRafNet^26^, DynGenie3^9^. When used on a single time series, the Random Forest for each gene is trained on all consecutive pairs of time points except the last time point. For example, if there are seven time points in the time series, then the Random Forest is trained based on the transitions from time point 1 to 2, 2 to 3, …, 5 to 6. Time point 7 will be predicted based on the trained function when applied to the data of time point 6. The net effect is that the testing points are not used in the training in any way because the test set includes only the last time points of each time series.

For a given time series, when multiple time series are available, OutPredict trains the Random Forest on all consecutive pairs of time points (always excluding the last time point) across all time series. Further, OutPredict treats replicates independently, viz. if there are k1 replicates for time point t1 and k2 for subsequent time point t2, then we consider k1 × k2 combinations in the course of our training. The result of the training is to construct a single function f for each target gene that applies to all time series. To test the quality of function f, we evaluate the mean-squared error (MSE) on the last point of every time series on that target gene.

The Random Forest uses bootstrap aggregation, where each new tree is trained on a sub-sample of the training data points. The Out-of-Bag error for a given training data point is estimated by computing the average difference between the actual value for a given training data point and the predictions based on trees that do not include the training data point in their bootstrap sample. Each tree is built on a bootstrap sample of size approximately 2/3 of the training dataset. Bootstrap sampling is done with replacement, and the remaining 1/3 of the training set is used to compute the out-of-bag score. Thus, the out-of-bag calculation is done on training data only.

All our experiments used random forest ensembles of 500 trees to avoid overfitting. Pruning did not improve the out-of-bag score, so the experiments used the default parameters for pruning of *RandomForestRegressor* in *sklearn*^27^.

### Incorporation of gold-standard data as priors

OutPredict uses prior data to bias the training of the Random Forest model. Specifically, each decision tree node within a tree of the Random Forest will be biased to include a transcription factor *X*_1_ for the model of gene *g* in preference to transcription factor *X*_2_ if the prior data indicates a relationship between *X*_1_ and g but none between *X*_2_ and *g*.

The gold standard for OutPredict is a matrix [Genes * TFs] containing 0 s and 1 s, which indicates whether we have prior knowledge about the interaction of a transcription factor (TF) and a gene. Hence, if the interaction between a TF and gene g is 1, then there is an inductive or repressive edge; while if it's 0, then there is no known edge.

In order to **compute prior weights** from the gold standard prior knowledge, we assign a value v to all interactions equal to 1 (i.e., the True Positive interactions) and 1/v to the interactions identified by 0 (the set of values tried for v is specified in Supplementary Table S2).

**Figure 1:**
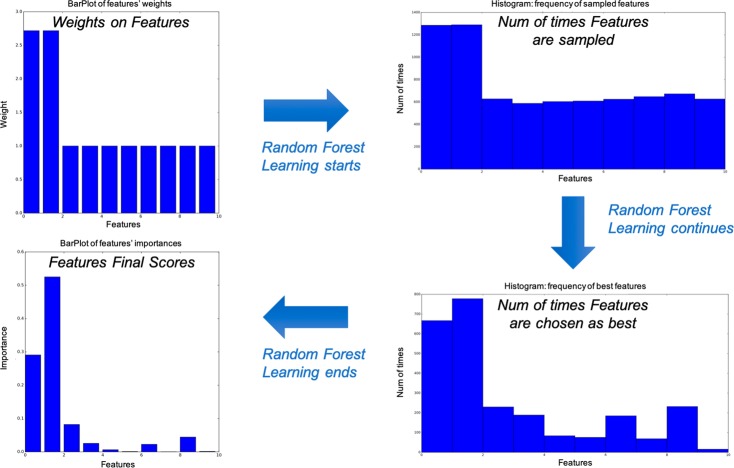
Illustration of how priors work: the priors assign initial weights to features (transcription factors) which influence how likely they are to be chosen as splitting elements in the trees of the Random Forest. As learning takes place, these weights can change, finally leading to a model that depends on both the time series data and on other data.

During the tree construction, our Weighted Random Forest, at each node *d*, selects *r* candidate features (transcription factors) *X*_1_, *X*_2_, …, *X*_*r*_ according to the prior weights (Fig. 1); *r* is the number of features sampled at each node *d*, which is set to the square root of the total number of transcription factors.

The r candidate transcription factors are a subset of all transcription factors and are randomly sampled at each tree node, biased based on the weights of the priors, as in iRafNet^26^. In addition, OutPredict calculates the *I*(*d*)(variance reduction * prior weight) criterion (which is defined below in formula (3) of the *Mathematical Formulation* section) for all the selected subset at each node and branch on the transcription factor with highest I(d).

OutPredict incorporates steady-state(SS) data into the same Random Forest model as the time series(TS) data (an “integrated“ approach, denoted as the *RF*_*SS*+*TS*_ model). Further, each prior dataset can be evaluated separately depending on how helpful it is to make predictions on time series. By contrast, for example, iRafNet^26^, combines all prior datasets and weights them equally at each tree node. An equal weighting strategy may decrease overall performance when, for example, one prior dataset is less informative or is error-rich. As an aside, iRafNet can make out-of-sample predictions but only on steady-state data.

### Mathematical formulation

Let *X* be the expression values of the set of features (in our case, transcription factors), and *y*_*j*_ be a target. We seek a function such that maps *X* to *y*_*j*_ either in steady-state or for time series. For steady-state data, we use all experimental conditions to infer a function *y*_*j*_ = *fsteady*_*j*_(*X*) where *X* must not include *y*_*j*_. That is, for each gene *y*_*j*_, we seek a function from all other genes to *y*_*j*_. For time series, Outpredict supports two types of models:

1. Time-Step (TS) model:

1 $${y}_{j}({t}_{i+1})=ftimeste{p}_{j}(X({t}_{i})),\forall j$$

2. Ordinary Differential Equation *natural* logarithm (ODE-log) model:

2 $$\frac{{y}_{j}({t}_{i+1})-{y}_{j}({t}_{i})}{\mathrm{ln}({t}_{i+1}-{t}_{i})}+\alpha {y}_{j}({t}_{i})=fod{e}_{j}(X({t}_{i})),\forall j$$

where *X*(*t*_*i*_) denotes the expression values of all the transcription factors at time *t*_*i*_, *y*_*j*_(*t*_*i*+1_) denotes the expression of gene *j* at *t*_*i*+1_, *α* is the degradation term. All genes are assumed to have the same *α*.

OutPredict integrates steady-state(SS) data with Time series(TS) data in a single Random Forest.

We have found that the ODE-log model achieves a better out-of-bag score compared to just using the linear difference (*t*_*i*+1_ − *t*_*i*_) in the denominator. This makes some intuitive sense because many phenomena in nature show a decay over time. Empirically, for example, the difference in expression value between 5 and 20 is more than 1/3 the difference between 5 and 60 in the Arabidopsis time series. Further, Supplementary Fig. S5 illustrates the absolute difference in gene expression decreasing over time for most of the species.

During training, one of the Time-Step or ODE-log models is selected based on the out-of-bag score on the training data. We have found that the relative performances of the two OutPredict techniques Time-Step and ODE-log are very data dependent, with Time-Step performing better than ODE-log on B. subtilis and Drosophila, while the opposite is observed on Arabidopsis, E.coli and DREAM4 (Supplementary Table S1 shows the best model based on out-of-bag score).

In detail, during training, OutPredict determines (i) which of these two methods (ODE-log or Time-Step) to use, (ii) the prior weights of the TFs, and (iii) the degradation term for the ODE-log model. As far as we know, this is the first time the choice of model and degradation parameter value have been treated as trainable hyper-parameters. We show in Supplementary Table S2 the set of hyper-parameter values tested for the degradation term *α* and for the prior weights when calculating the out-of-bag score.

Computationally, at a given node d in a tree, OutPredict computes the product of (i) the standard Random Forest importance measure which is defined as the total reduction of the variance of y and (ii) the weight given by the priors. Here is the formula used for the reduction of variance^8^, modified by the prior weighting:

3 $$I(d)=[({S}_{num}\ast {\mathrm{var}}_{y}(S))-({S}_{{l}_{num}}\ast {\mathrm{var}}_{y}({S}_{l}))-({S}_{{r}_{num}}\ast {v}_{y}({S}_{r}))]\ast {w}_{{X}_{i},y}$$

where *d* is the current decision node being evaluated, *S* is the subset of samples that are below decision node d in the tree, *S*_*l*_ and *S*_*r*_ are the subsets of experiments on the left and right branches of decision node d, respectively; *var*_*y*_ is the variance of the target gene in a given subset, and ${S}_{num},{S}_{{l}_{num}},{S}_{{r}_{num}}$ denote the number of training samples in each subset associated with a specific target gene. Finally, ${w}_{{X}_{i},y}$ is the prior weight from a given feature *X*_*i*_ to a given target gene *y*, which causes features with high prior weights to be chosen with higher probability when splitting a tree node during tree construction. Because the model for each target gene is independent, OutPredict calculates the model for the target genes in parallel.

For the purpose of inferring relative influence of transcription factors on genes and constructing a network of such potential causal edges, let *T* be the number of trees and *D*_*i*_ be the set of nodes which branch based on transcription factor (feature) *X*_*i*_, the overall importance score of the feature *X*_*i*_ is:

4 $${s}_{i}=\frac{1}{T}\sum _{{D}_{i}}\,I(d)$$

Computationally, the importance score *s*_*i*_ of *X*_*i*_ is the sum of the variance improvements I(d) over all nodes d in *D*_*i*_ divided by the number of trees T. The resulting variable importance value *s*_*i*_ is more robust than the value obtained from any single tree because of the variance reduction resulting from averaging the score over all the trees^8^. High importance scores identify the set of the likely most influential transcription factors for each target gene.

## Results

We measure the prediction performance of our algorithm using the Mean Squared Error(MSE) of the predictions of out-of-sample data. For each species tested, we compare the performance of the different algorithms on time series alone and on time series data with prior information.

As mentioned above, we compared our weighted Random Forest with two related works: (i) a Neural Network (NN) with a hidden layer^13^ which is an approach developed specifically for time series gene expression prediction (in the supplement). In detail, we perform hyper-parameter optimization for the learning rate of the stochastic gradient descent optimizer, and the dropout rate. Thus, regularization is applied through dropout, which helps reduce overfitting. (ii) the Random Forest algorithm DynGenie3^9^, which is an extension of Genie3^25^ that is able to handle both steady-state and time series experiments through the adaptation of the same ordinary differential equation (ODE) formulation as in the Inferelator approach^6^. iRafNet^26^, as noted above, does not handle time series data as the main input data.

**Algorithm 1:**
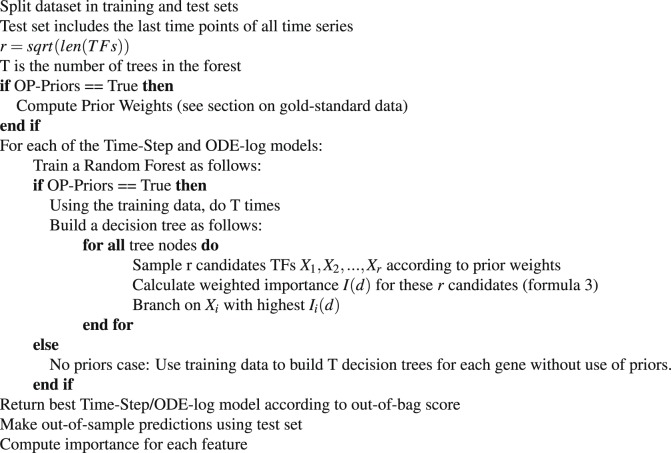
OutPredict method.

**Figure 2:**
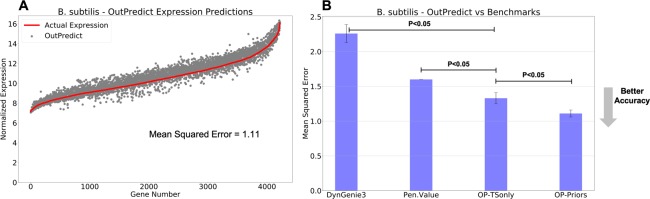
Bacillus subtilis. (**A**) Comparison of predicted gene expression using OutPredict (grey dots) versus actual expression (red line) at the left-out time point. Genes are ordered by increasing actual mean expression value (red line). OutPredict predicts gene expression well at all expression levels. The accuracy of forecasting is measured by calculating the Mean Squared Error (MSE). (**B**) The vertical axis indicates MSE, where lower bars indicate more accurate predictions. The descriptions of the different models of the x axis can be found in Table 2. OutPredict (*OP-Priors*) performs significantly better (P < 0.05, based on a non-parametric paired test) than *Penultimate Value* (with a 30% relative improvement), DynGenie3 (with a 50% relative improvement) and Neural Network(NN). The MSE for Neural Nets is 3.75 (with standard deviation ≈0.3), which is considerably higher than for other methods (Supplementary Table S3); it is not shown here because the MSE is out of scale. Moreover, when priors from both Integrated steady-state data and prior gold standard data, are used with the OutPredict algorithm, there is a significant (P < 0.05, non-parametric paired test) improvement in predictions relative to OutPredict using only time series data. Specifically, prior gold standard data is significantly helpful, showing a 11% relative improvement (Supplementary Fig. S4). Finally, out-of-bag analysis concludes that the Time-step differencing model is better than the ODE-log.

**Figure 3:**
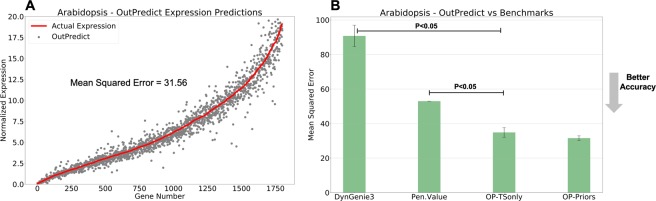
Arabidopsis in Shoot Tissue (time series only dataset) (**A**) Predicted gene expression using OutPredict (grey dots) compared to actual expression (red line) at the left-out time point. (**B**) Comparison of time series forecasting: the accuracy of forecasting, measured by Mean Squared Error, has higher values in this case than for other species, because the data is RNAseq and read counts have a broad dynamic range. Table 2 describes which method and data were used for each model in the x axis. OutPredict (*OP*) performs 34.2% better than *Penultimate Value* (P < 0.05, non-parametric paired test), and 61.5% better than Dynamic Genie3 (P < 0.05, non-parametric paired test). The incorporation of priors from *TARGET* (*OP-Priors*) improves the performance of OutPredict compared to the time series alone (9% improvement with P = 0.12, non-parametric paired test). The ODE-log model is better than Time-Step based on the out-of-bag score. The Neural Network model doesn’t converge because the dataset is small.

DynGenie3 was primarily designed for Gene regulatory network inference, but the authors show the performance of DynGenie3 at predicting both time series and steady-state data in the validation sets. Therefore, we evaluate DynGenie3 for predicting leave-out time series data in order to compare it with OutPredict. As a baseline for all algorithms, we consider the *penultimate value* prediction of the expression of a gene at a given time point to be the same value as the expression of that gene at the immediately previous time point. To evaluate the performance of our forecasting predictions, we compare the predicted expression values to the actual expression values for each gene (Figs. 2A, 3A) and calculate the Mean Squared Error (MSE) across all genes.

### Quantitative results

We show in Figs. 2B and 3B overall bar plots for a *Bacillus subtilis* and *Arabidopsis*. Similar results hold for other species (Supplementary Figs S1, S2, S3). A table showing which method and data were used for each can be found in Table 2. Our basis of comparison is Mean Squared Error, which is a measure of the error in the predictions in which smaller values indicate more accurate predictions. Given a species, the mean squared error (MSE) is calculated as follows: given the prediction and actual value for each replicate of each gene at the last time point, first compute the squared error for each replicate. Second, take the mean to get the mean squared error for that gene. Third, compute the global mean squared error as the mean of the mean squared errors of each gene. Figures 2A and 3A show qualitatively that the actual values closely track the predicted values. OutPredict outperforms DynGenie3, Neural Nets, and *penultimate value* predictions over all species using these datasets.

**Table 2:** Legend of Experimental Results.

Label	Method	Description
OP-Priors	OutPredict-Priors	OutPredict uses (i) Time series(TS) with steady-state(SS) data integrated (TS + SS) in one big Random Forest, and (ii) Gold standard data as priors to bias the integrated Random Forests for time series and steady-state data.
OP-TSonly	OutPredict-TimeSeriesOnly	No Priors: Time series alone; no other data.
DynGenie3	Dynamic Genie3	settings and hyper-parameter optimization as described in^9^
NN	Neural Network	one hidden layer as described in^13^
Pen. Value	Penultimate Value	the second to last time points of each time series is used as the prediction for the last one.

In B. subtilis (Fig. 2), OutPredict performs 30% better than Penultimate Value (P < 0.05, based on a non-parametric paired test), and 50% better than Dynamic Genie3 (P < 0.05, based on a non-parametric paired test) (Fig. 2B). As OutPredict allows the incorporation of priors into the model, such as gold-standard network data, we compared the forecasting performance of OutPredict using time series with the integration of steady-state with OutPredict on time series data with steady-state data and gold-standard regulated edges as priors (Supplementary Fig. S4). In these tests, the inclusion of validated gold-standard edges as priors improved predictions compared to excluding priors (Supplementary Fig. S4, 11% improvement, P < 0.05, non-parametric paired test).

The non-parametric paired test we use throughout this paper compares any two prediction methods M1 and M2 as follows: (i) format the data from the original experiment by a series of rows with one row for each gene containing the gene identifier, the M1 prediction for that gene, the M2 prediction, and the real value (call this series of rows *Orig*); (ii) calculate the figure of merit (for example, the squared error) for each gene and each method (e.g., the square of M1 prediction - real value); (iii) calculate the difference, *Diff*, in the average of the figure of merit (for example, the difference of the mean squared errors) of the M1 values and the M2 values; (iv) Without loss of generality, assume *Diff* is positive; (v) randomization test: for some large number of times N (e.g., N = 10,000), starting each time with *Orig*, for each gene g, swap the M1 and M2 values for gene g with probability 0.5. Now recalculate the overall difference of the figure of merit for M1 and for M2 and see if that difference is greater than *Diff*. If so, that run is considered an *exception*; (vi) The p-value of *Diff* (and therefore of the change in the figure of merit) is the number of exceptions divided by N. When the p-value is small, the observed difference is unlikely to have happened by chance.

We show in Table 2 the different models that were compared for the experimental results: each model (built with a given algorithm) is associated with a given species, a specific main input dataset and a prior dataset. Recall that, in OutPredict, the priors bias the Random Forest by adjusting the weights that determine feature inclusion.

Furthermore, we show the results using the OutPredict (*OP*) technique (either the Time-step or ODE-log) that validation analysis found to be the best model using the out-of-bag score. We found that the weights/importance found in high quality prior data significantly improve predictions in B. subtilis (Fig. 2B), though less so in Arabidopsis Shoots (Fig. 3B). There is no improvement in E. coli, Drosophila or Dream4 (Supplementary Figs S1, S2, S3). The precise reasons may vary: gold standard data may contain inaccurate regulatory interactions, may be either incomplete, or may depend on specific experimental conditions. The DREAM4 dataset shows that Priors data contributes to out-of-sample predictions more when there are few time series than when there is abundant time series data (Supplementary Fig. S8); similarly, the out-of-sample predictions improvement of using time steady-state data, relative to time series data alone, decreases as the number of time series increases (Supplementary Fig. S7).

**Figure 4:**
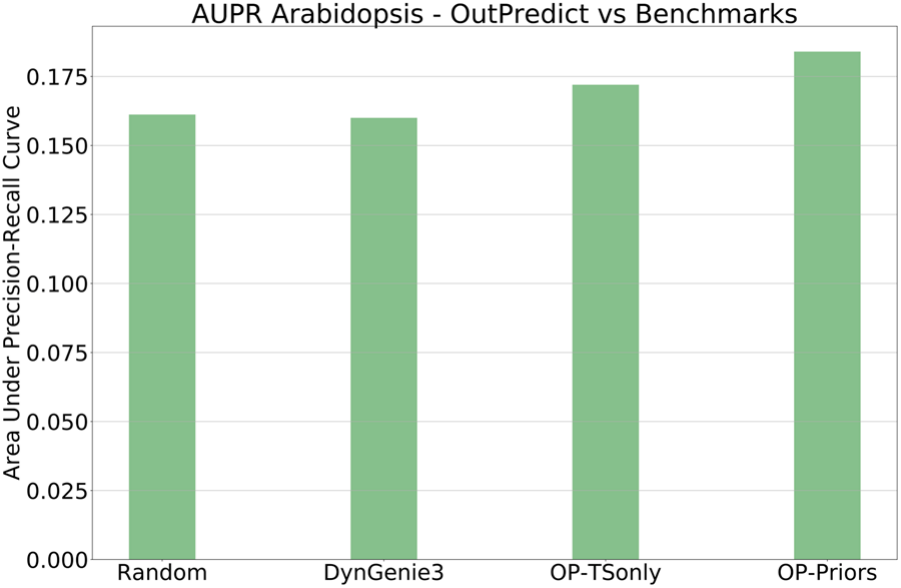
Inference of Causality. The area under the precision recall curve (AUPR) of Outpredict with Priors (OP-Priors) is 15% better than random (p-value < 0.01, based on a non-parametric paired test); AUPR of Outpredict without Priors (OP-TSonly) is 7.5% better than random (p-value < 0.01, non-parametric paired test); DynGenie3 same as random.

As a test of the usefulness of OutPredict's importance scores, or measures of influence, for all the TFs on every target gene, we evaluate the *OP-Priors* model importances in Arabidopsis. The dataset consists of 162 TFs on 2173 targets, totaling 352,026 TF–target edges. To refine these time-based TF–target predictions, we retained the highest-confidence edges, specifically, the top 2% of the edges according to the score, resulting into 7042 edges. We used 1754 validated TF–target edges of 11 TFs physical experiments from^28–35^, (the data for the 11 TFs are described in Supplementary Table S4), which is a disjoint dataset from the one used for the priors. This analysis establishes the precision (i.e., the proportion of predicted TF-target edges that are validated) and recall (i.e., the proportion of validated TF-target edges that are predicted) of the OutPredict top 2% edges for the validated 11 TFs. The results showed that precision and recall for the TF–target predictions in the top 2% edges were 0.246 (76/309) and 0.043 (76/1754), respectively. Both were significantly greater than the mean for 1000 random samples of 309 edges of these 11 TFs (random precision mean ≈0.161 and random recall mean ≈0.028) (Table 3). Moreover, the precision of OP-Priors for the top 2% outperforms OP-TSonly (precision = 0.226) and DynGenie3 (precision = 0.158). We further compared the performance of the OP-Priors model importances with OP-TSonly and DynGenie3, and computed the Area under Precision-Recall (AUPR) using the 1754 validated TF–target edges of 11 TFs physical experiments in Arabidopsis. The AUPR of Outpredict with Priors (OP-Priors) is 15% better than random (p-value < 0.01, non-parametric paired test), for Outpredict without Priors (OP-TSonly) AUPR is 7.5% better than random (p-value < 0.01, non-parametric paired test), while DynGenie3 is no better than random (Fig. 4). In the supplement (Supplementary Fig. S9), we show that similar results hold for the DREAM4 synthetic dataset (where causal edges are known). This shows the promise of using prediction to infer influence and suggests that good out-of-sample prediction leads to good causality models.

**Table 3:** TF-target validation for *OP-Priors* Arabidopsis Model.

Validated TF-target measures	OP-Priors
Precision/Recall TF-target	0.246/0.043
Random Precision/Recall average	0.161/0.028
Validated Precision/Recall p-value	<0.01/<0.01

The important edges predicted by the model had a precision and recall of over 23% and 4%, respectively. Whereas a random selection of the same number of edges had a precision and recall of 16% and under 3% (respectively). The differences for both are statistically significant.

## Discussion

OutPredict is a non-linear machine learning method based on an ensemble of regression trees for time series forecasting. It can incorporate steady-state data, temporal data and prior knowledge, as well as a variety of differential equation models for this purpose. OutPredict both predicts the future states of a given organism and gives a quantitative measure of the importance of a given transcription factor on a target gene.

There are four reasons for the relative success of OutPredict compared to other methods: (i) the use of Random Forests which provides a non-linear model (in contrast to regression models) that requires little data (in contrast to neural net approaches), (ii) the incorporation of prior information such as gold standard network data (in contrast to DynGenie3), (iii) the adjustment of weights of predictors (in contrast to all other time series based methods), and iv) the selection during training of the optimal technique between the Time-Step and our *ODE-log* model, which includes a degradation term that is also tuned (in contrast to all other methods).

In summary, OutPredict achieves high prediction accuracy and significantly outperforms baseline and state-of-the-art methods on data sets from four different species and the in silico DREAM data as measured by mean squared error. Further, as a proof of concept, we have seen that the high importance edges correspond to individually validated regulation events much greater than by chance in both Arabidopsis and DREAM. The code is open source and is available at the site https://github.com/jacirrone/OutPredictgithub.com/jacirrone (10.5281/zenodo.3611488).

## Supplementary information

Supplementary Information.
