# Author Correction: OutPredict: multiple datasets can improve prediction of expression and inference of causality

**DOI:** 10.1038/s41598-020-69883-2

**Published:** 2020-08-19

**Authors:** Jacopo Cirrone, Matthew D. Brooks, Richard Bonneau, Gloria M. Coruzzi, Dennis E. Shasha

**Affiliations:** 1 Courant Institute of Mathematical Sciences, Department of Computer Science, New York University, New York, NY 10012 USA; 2 Center for Genomics and Systems Biology, Department of Biology, New York University, New York, NY 10003 USA; 3 Center for Computational Biology, Flatiron Institute, Simons Foundation, New York, NY 10010 USA

Correction to: *Scientific Reports* https://doi.org/10.1038/s41598-020-63347-3, published online 22 April 2020

The Acknowledgements section in this Article is incomplete.

“The authors gratefully acknowledge funding from the following sources: NIH NIGMS Grant GM032877 to G.M.C. and D.E.S., NSF-PGRP IOS-1339362 to G.M.C. and D.E.S., an NIH NIGMS Fellowship 1F32GM116347 to M.D.B., and a Plant Genomics Grant from the Zegar Family Foundation (A160051).”

should read:

“The authors gratefully acknowledge funding from the following sources: NIH RO1 GM121753 to G.M.C. and D.E.S., NIH NIGMS Grant GM032877 to G.M.C. and D.E.S., NSF-PGRP IOS-1339362 to G.M.C. and D.E.S., an NIH NIGMS Fellowship 1F32GM116347 to M.D.B., and a Plant Genomics Grant from the Zegar Family Foundation (A160051).”

