# Supplementary material for: OutPredict: multiple datasets can improve prediction of expression and inference of causality
Source: Sci Rep. 2020 Apr 22;10:6804. doi: 10.1038/s41598-020-63347-3 (PMC7176633; doi:10.1038/s41598-020-63347-3)
Supplement: Supplementary file 1 — Supplementary Information. [file 41598_2020_63347_MOESM1_ESM.pdf]

OutPredict: multiple datasets can improve prediction of expression and inference of causality
Supplementary Information

Jacopo Cirrone, Matthew D. Brooks, Richard Bonneau, Gloria M. Coruzzi, and Dennis E. Shasha
New York University, New York, USA

1 Supplementary Figures

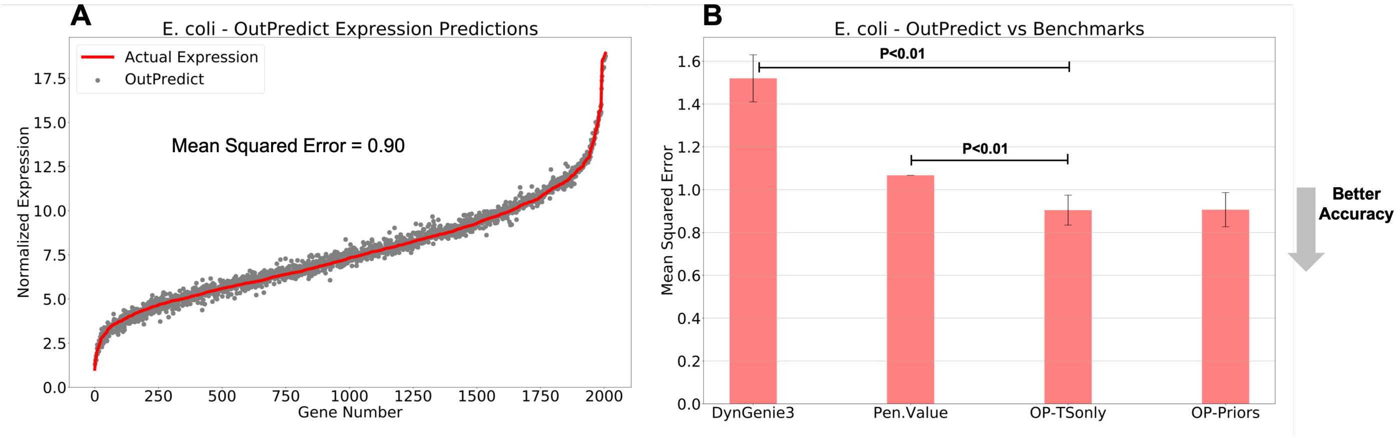

Figure S1: **Escherichia coli: Time series forecasting.** This is a time series only dataset consisting of 15 time series. (A) Comparison of predicted gene expression using OutPredict (grey dots) vs. actual expression (red line) at the left-out time point. The accuracy of forecasting is measured by calculating the Mean Squared Error. (B) OutPredict (*OP* and *OP-Priors*) improves ($P < 0.01$, based on a non-parametric paired test) the quality of forecasting compared to *Penultimate Value* (15% improvement) and Dynamic Genie3 (40.5% improvement). For this data, there is no improvement using priors from gold-standard edges compared with time series data by itself.

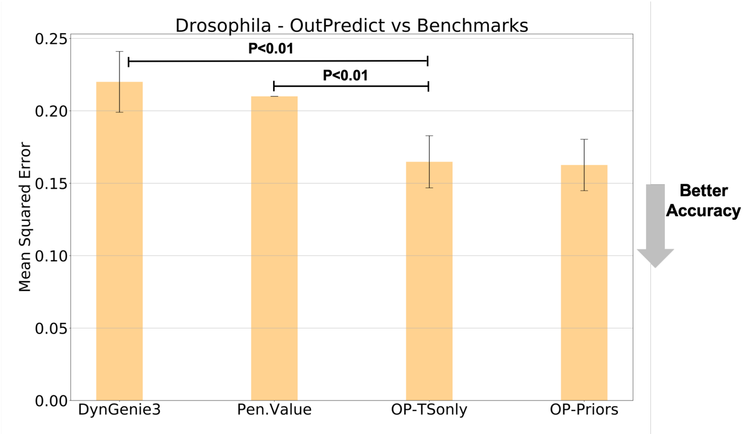

Figure S2: **Drosophila: Time series forecasting.** This is a time series only dataset consisting of one time series of 28 time-points. OutPredict (*OP* and *OP-Priors*) performs better (P $<$ 0.01, non-parametric paired test) than benchmark approaches including *Penultimate Value* and Dynamic Genie3 (23% and 26.1% improvement, respectively). The incorporation of priors from the gold-standard network does not improve forecasting compared to time series alone.

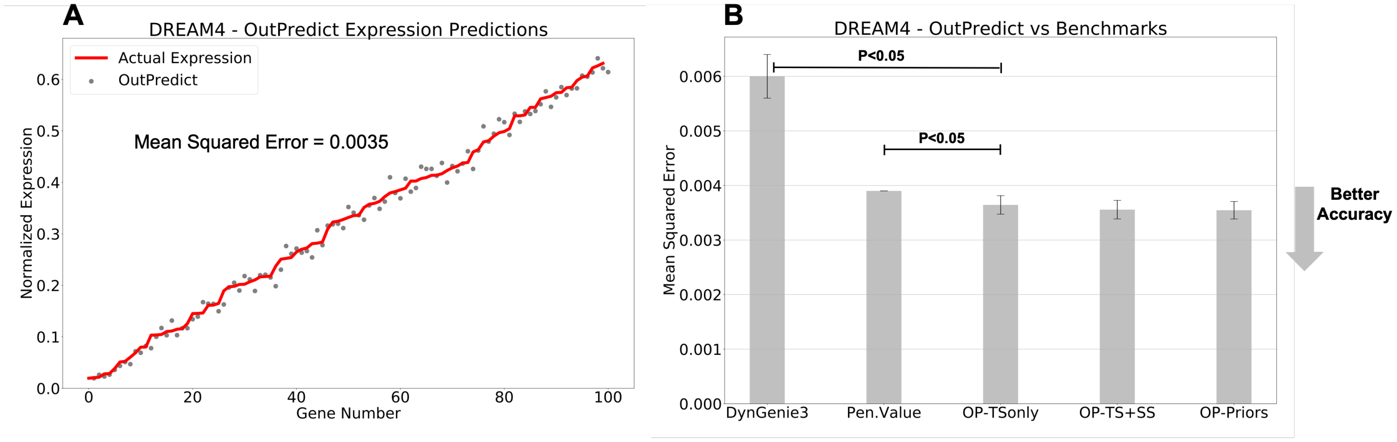

Figure S3: **DREAM4: Time series forecasting.** This is a synthetic dataset. (A) Comparison of predicted gene expression using OutPredict (grey dots) vs. actual expression (red line) at the left-out time point. (B) OutPredict (*OP-TSonly*, *OP-TS+SS* and *OP-Priors*) outperforms (P $<$ 0.05, non-parametric paired test) *Penultimate Value* and Dynamic Genie3 with 10% and 40.1% relative improvement, respectively. The incorporation of priors together with the integration of steady-state data does not improve forecasting compared to time series alone.

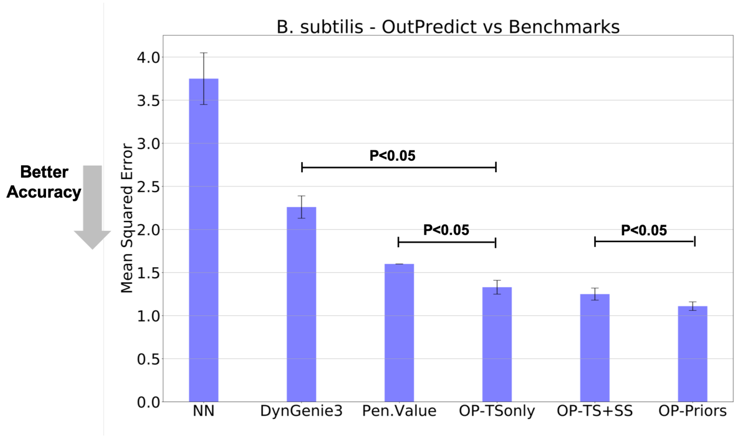

Figure S4: - Bacillus Subtilis - Full Comparison of time series forecasting: Neural Network from [Smith et al 2010] (NN) vs. Dynamic Genie3 (DynGenie3) vs. Penultimate Value (Pen.Value) vs. OutPredict (*OP-TSonly*, *OP-TS+SS* and *OP-Priors*). The use of steady-state data (*OP-TS+SS*) leads to a 6% significant improvement ($P < 0.05$, non-parametric paired test) relative to time series data alone (*OP-TSonly*). *OP-Priors* uses gold standard data (in addition to time series (TS) and steady-state (SS) integrated in a single random forest), which is helpful compared to the model *OP-TS+SS* showing an 11% relative improvement ($P<0.05$, non-parametric paired test).

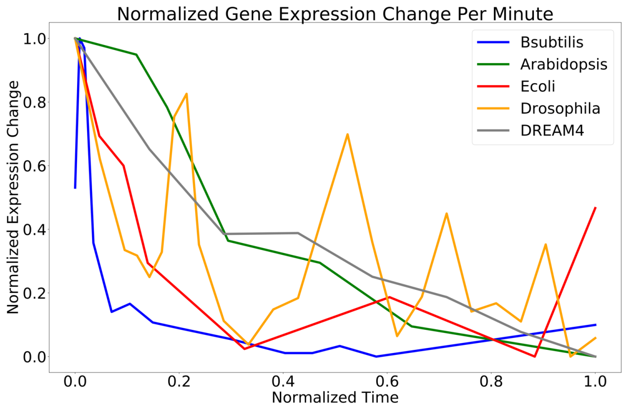

Figure S5: Gene Expression Change for all species. Generally, the average absolute difference in expression (across all genes for each species) decreases over time. E. Coli may be an exception because of the short lifespan of bacteria. The Time-Step model worked better for B. subtilis and Drosophila. The Ordinary Differential Equation-log model worked better for Arabidopsis, E. coli and DREAM4 (Supplementary Table S1).

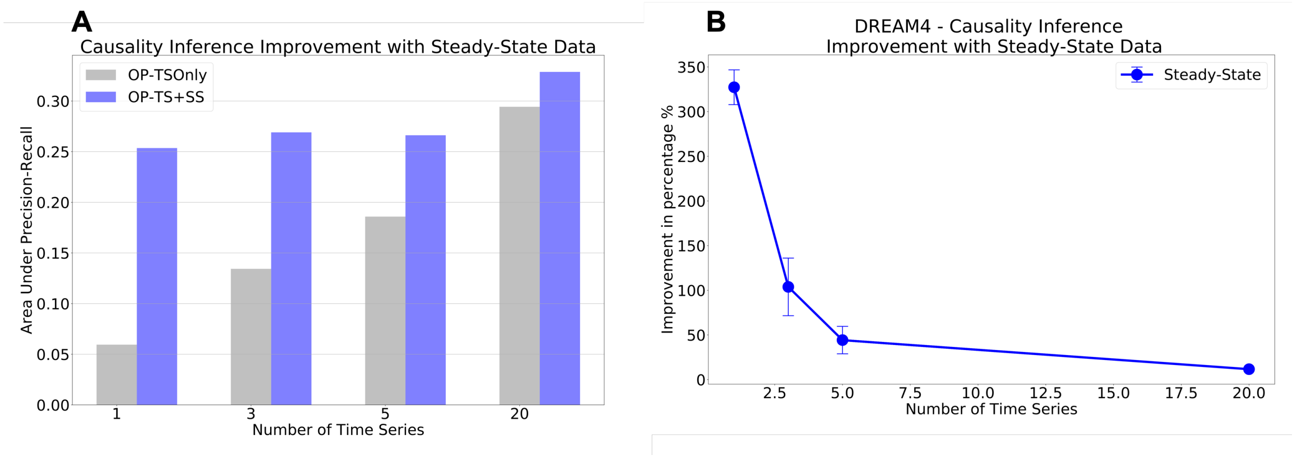

Figure S6: DREAM4 - Causality Inference Improvement with Steady-State data. The DREAM4 dataset shows that steady-state data contributes to the inference of causality more when there are few time series than when there is abundant time series data. (A) We show the comparison of Area under Precision-Recall (AUPR) with and without steady-state data in cases of different numbers of time series. The y-axis represent the AUPR average of three different random sets of time series of size $1, 3, 5$ respectively; $x = 20$ represents the case of taking all 20 time series in the DREAM4 dataset. (B) The AUPR improvement of using time steady-state data, relative to time series data alone, decreases as the number of time series increases.

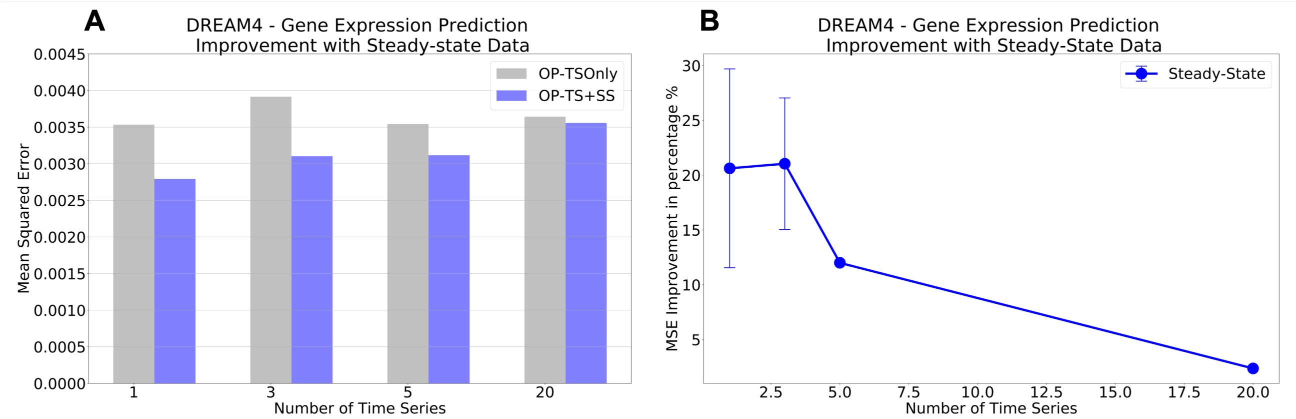

Figure S7: DREAM4 - Gene Expression Prediction Improvement with Steady-State data. The DREAM4 dataset shows that steady-state data contributes to out-of-sample prediction more when there are few time series than when there are many. (A) We show the comparison of time series forecasting with and without steady-state data for different numbers of time series. The y-axis represent the MSE (mean squared error) average for three different random sets of time series of sizes $1, 3, 5$ respectively; $x = 20$ represents the use of all 20 time series in the DREAM4 dataset. (B) The out-of-sample predictions improvement of using time plus steady-state data, relative to time series data alone, decreases as the number of time series increases.

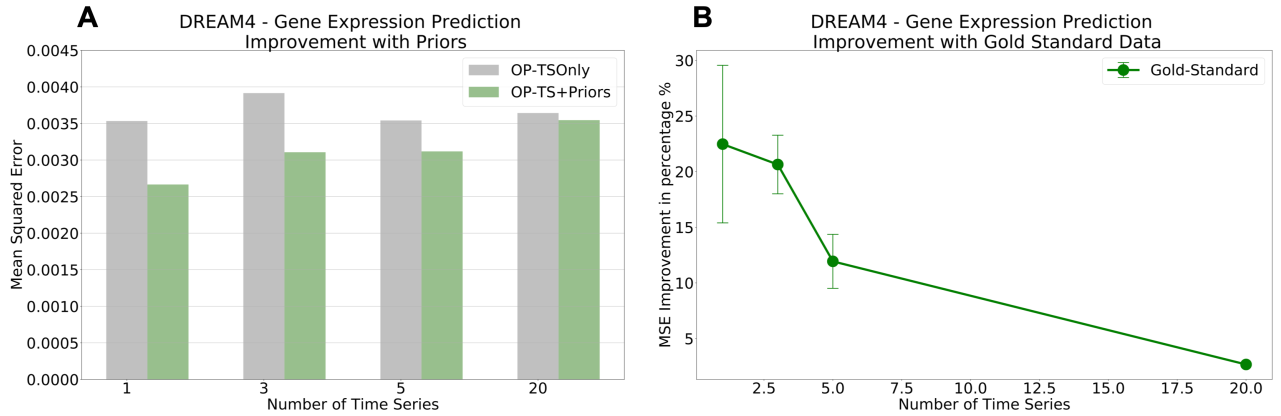

Figure S8: DREAM4 - Gene Expression Prediction Improvement with Priors. The DREAM4 dataset shows that Priors data contributes to out-of-sample predictions more when there are few time series than when there are many. (A) We show the comparison of time series forecasting with and without gold standard data for different numbers of time series. The y-axis represent the MSE (mean squared error) average for three different random sets of time series of size $1, 3, 5$ respectively; $x = 20$ represents the use of all 20 time series in the DREAM4 dataset. (B) Therefore, when the gold standard as priors is used in addition to time series data, the out-of-sample prediction improvement decreases as the number of time series increases.

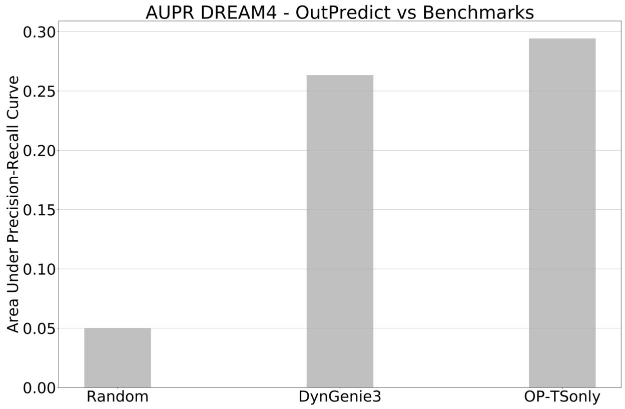

Figure S9: AUPR DREAM4 - OutPredict vs. Benchmarks for the inference of causal edges. As for the Arabidopsis dataset (Figure 4 of the main paper), here we show the AUPR (Area Under the Precision-Recall curve) for predicting causal edges in the ideal case of DREAM4 where the true gold standard is known. Outpredict without Priors (OP-TSonly) is clearly better than random (p-value < 0.01, non-parametric paired test) in terms of Area under Precision-Recall. Further, AUPR of OP-TSonly is 12% better than AUPR of DynGenie3 on time series data (p-value < 0.01, non-parametric paired test). This suggests that good out-of-sample prediction leads to good causality models.

Dataset	Best OutPredict Model
B. subtilis	Time-Step (7% better than ODE-log)
Arabidopsis	ODE-log (22% better than Time-Step)
E. coli	ODE-log (15% better than Time-Step)
Drosophila	Time-Step (17% better than ODE-log)
DREAM4	ODE-log (5% better than Time-Step)

Table S1: Time-Step(TS) vs ODE-log model. For a given organism the table shows the best model based on out-of-bag score. The relative performance of the two OutPredict techniques Time-Step and ODE-log are very data dependent, with Time-Step performing better than ODE-log on B. subtilis and Drosophila, while the opposite is observed on Arabidopsis, E.coli and DREAM4. We determine this on the training data and then apply whichever method is better on the test data.

Hyper-parameter	Set of values tested
alpha (α)	[1, $2e{-}1$, $1e{-}1$, $4e{-}2$, $2e{-}2$, $1e{-}2$]
prior weights (True Positive)	[2,exp(1), 5, 8, 15]

Table S2: Hyper-parameters: Set of values tested for the degradation term alpha (α) and for the prior weights when calculating the out-of-bag score. As explained in the body of the paper, when OP-Priors is set to $True$ and gold standard data is given as priors, OutPredict transforms the gold standard prior knowledge to prior weight, by assigning a value v (chosen from the set of prior weights in the table) to all interactions where there is an edge in the prior data and 1/v to the interactions where the existence of an edge is unknown.

Dataset	Neural Netwok MSE (StdDev)	OutPredict Time-Series-only MSE (StdDev)
B. subtilis	3.75 (0.3)	1.33 (0.08)
E. coli	3.33 (0.27)	0.9044 (0.07)
DREAM4	0.0095 (0.0008)	0.0036(0.00017)

Table S3: Neural Network (NN) with one hidden layer [Smith et al 2010] vs. OutPredict Time-Series-only (OP-TSonly). NN from [Smith et al 2010] is able to learn using time series only datasets. The table shows that the mean squared error (MSE) for NN is significantly higher than for OutPredict since there is a relatively small amount of data. Neural Networks work best with much larger datasets. NN doesn't converge for Arabidopsis and Drosophila because the datasets are too small.

**Transcription Factor**	**Technology**
CGA1/GNL(AT4G26150)	Microarray-Agilent
GATA17(AT3G16870)	Microarray-Agilent
GATA2(AT2G45050)	Microarray-ATH1
LBD38(AT3G49940)	Microarray-ATH1
LBD37(AT5G67420)	Microarray-ATH1
PHR1(AT4G28610)	Microarray-ATH1
NLP7(AT4G24020)	Microarray-CATMA
HBI1(AT2G18300)	RNA-seq
CRF4(AT4G27950)	RNA-seq
GNC(AT5G56860)	Microarray-Agilent<br>combined with RNA-seq experiment
SVP(AT2G22540)	RNA-seq

Table S4: The Transcription Factor (TF) experiments used for the validation of OutPredict's Arabidopsis Model importance output. Regarding the Microarray experiments, the genes not on chip were filtered from the predictions according to the microarray type. The microarray elements for the different types were retrieved from the following public repository: CATMA in arabidopsis.org ; ATH1 in arabidopsis.org ; Agilent in arabidopsis.org.
